# Invasive cervical cancers in the United States, Botswana and Kenya: HPV type distribution and health policy implications

**DOI:** 10.1186/s13027-016-0102-9

**Published:** 2016-11-11

**Authors:** Aaron Ermel, Brahim Qadadri, Yan Tong, Omenge Orang’o, Benson Macharia, Doreen Ramogola-Masire, Nicola M. Zetola, Darron R. Brown

**Affiliations:** 1 Indiana University School of Medicine, Indianapolis, IN USA; 2 Moi University and Moi Teaching and Referral Hospital, Eldoret, Kenya; 3 Botswana-University of Pennsylvania Partnership, Gaborone, Botswana

## Abstract

**Background:**

More deaths occur in African women from invasive cervical cancer (ICC) than from any other malignancy. ICC is caused by infection with oncogenic types of human papillomavirus (HPV). Co-infection with the human immunodeficiency virus (HIV) accelerates the natural history of ICC, and may influence the HPV type distribution. Because HPV vaccines are available, this malignancy is theoretically preventable, but the vaccines are largely type-specific in protection against infection. Data on specific HPV types causing ICC in African women is limited, and many studies utilized swab samples rather than actual cancer tissue. A previous study using archived, ICC tissue from women in Botswana identified an unusual HPV type distribution. A similar study was therefore performed in a second sub-Saharan country to provide additional information on the HPV type distribution in ICC.

**Methods:**

Archived, formalin-fixed, paraffin-embedded ICCs were acquired from women in the United States, Kenya, or Botswana. DNA was extracted and HPV genotyping performed by Roche Linear Array. HIV sequences were identified in ICCs by PCR.

**Results:**

HPV types 16 or 18 (HPV 16/18) were identified in 93.5 % of HPV-positive ICCs from the U.S., 93.8 % from Kenya, and 61.8 % from Botswana (*p* < 0.0001). Non-HPV 16/18 types were detected in 10.9 % of HPV-positive cancers from the U.S., 17.2 % from Kenya, and 47.8 % from Botswana (*p* < 0.0001). HIV was detected in 2.2, 31.5, and 32.4 % from ICCs from the U.S., Kenya, or Botswana, respectively (*p* = 0.0002). The distribution of HPV types was not significantly different between HIVinfected or HIV-uninfected women. The percentages of ICCs theoretically covered by the bivalent/quadrivalent HPV vaccines were 93.5, 93.9, and 61.8 % from the U.S., Kenya and Botswana, respectively, and increased to 100, 98, and 77.8 % for the nanovalent vaccine.

**Conclusions:**

HPV 16/18 caused most ICCs from the U.S. and western Kenya. Fewer ICCs contained HPV 16/18 in Botswana. HIV co-infection did not influence the HPV type distribution in ICCs from African women from the two countries. Available HPV vaccines should provide protection against most ICCs in the U.S. and Kenya. The recently developed nanovalent vaccine may be more suitable for countries where non-HPV 16/18 types are frequently detected in ICC.

**Electronic supplementary material:**

The online version of this article (doi:10.1186/s13027-016-0102-9) contains supplementary material, which is available to authorized users.

## Background

An estimated 528,000 cases of invasive cervical cancer (ICC) occurred worldwide in 2012 [1]. More deaths occur in African women from cancer of the cervix than from any other malignancy. The highest incidence rates of ICC occur in Eastern and Southern Africa, with age standardized rates (ASR) of 42.7 and 31.5 per 100,000 women, respectively [1].

Many obstacles exist for sub-Saharan countries in the fight against cervical cancer, where this malignancy is diagnosed most often at ages 35 to 45 years, claiming the lives of women when they may be raising children and contributing to the economy of their communities [2]. All but the least progressed stages of cervical cancer have high case-fatality rates in this region of the world (estimated to be greater than 50 %) due to poor access to effective treatment modalities. Women with advanced cases have 2-year survival rates of less than 20 % [3], and the average life-years lost due to cervical cancer is greater than 18 [4]. Effective screening programs utilizing cervical cytology have decreased the number of cases of ICC in developed nations. However, screening is rarely performed in sub-Saharan Africa, where more than 50 % of global cervical cancer deaths occur.

Infection with oncogenic human papillomaviruses (HPV) is the cause of ICC [5]. Cervical cancer can be prevented by vaccination against HPV infection, but the distribution of HPV types in invasive cancers has not been fully characterized, especially in women infected with the human immunodeficiency virus (HIV) [6].

As sub-Saharan African countries consider implementing HPV vaccination, it is important to understand the range of HPV types responsible for ICC cases in a country. Surveillance and better understanding of local epidemiology must be encouraged before selecting the HPV vaccine of choice for a specific country. The object of the study was therefore to compare HPV typing data from a previously published study of ICC cases in the United States and Botswana [7] to additional cases identified in a second sub-Saharan country, Kenya, and to determine the HPV type distribution in invasive cervical cancers from HIV-infected women living in these countries.

## Methods

### Human subject approval

Approval for the study was granted by the Institutional Review Board at Indiana University School of Medicine, University of Pennsylvania, the Botswana Ministry of Health, Gaborone, Botswana, and by the Institutional Research and Ethics Committee at Moi University Teaching and Referral Hospital. All specimens were de-identified prior to transport to the HPV Laboratory.

### Study specimens

Acquisition of archived, paraffin-embedded blocks of formalin-fixed cervical cancer specimens from women residing in Indianapolis, Indiana (U.S.) and from Botswana was previously described [7]. Case selection was done by working backwards in time among archived paraffin blocks. All ICC specimens from the U.S. were obtained from the Department of Pathology at Indiana University School of Medicine.

Additional ICC specimens, obtained from 2010 to 2013 were identified at the Moi University Teaching and Referral Hospital in Eldoret, Kenya. Section (5 μm) from paraffin-embedded specimens were cut using a new blade for each block. The first and last sections were stained with hematoxylin and eosin for verification of ICC. Specimens without invasive cancer tissue were eliminated from the study.

The remainder of each block was transported to Indiana University School of Medicine (Indianapolis), where tissues were re-embedded due to apparent dehydration of paraffin. Additional sections were prepared, and the first and last sections were stained with hematoxylin and eosin to verify the presence of ICC, Those without invasive cancer tissue were eliminated from the study. For specimens containing cancer tissue representing less than an estimated 5 % of the total specimen, macro-dissection was performed by identifying cancerous tissue using a light microscope, marking the areas cancer tissue on the undersurface of the slide, and removing this tissue with a sterile scalpel.

### HPV detection and typing

Thin-section polymerase chain reaction (PCR) of specimens from Kenyan women was performed exactly as described in a prior report of ICC cases from women living in Botswana and the U.S. [7]. Briefly, four to six paraffin-embedded tissue sections were scraped from slides prepared from each block using a sterile blade. DNA extraction was then performed as previously described [7].

HPV detection and genotyping was performed using the Linear Array HPV Genotyping Test (Roche, Indianapolis, Indiana) as described [8]. The GH20/PC04 human β-globin target was co-amplified to determine specimen adequacy. For specimens positive for β-globin but negative for HPV, DNA was extracted a second time from additional sections. If the specimen was negative for β-globin a second time, it was excluded in the analysis. If the specimen was positive for β-globin but negative for HPV a second time, it was considered to be HPV-negative.

### Detection of HIV

Sera were not available from women, as this study used archived ICC specimens. In addition, medical records were not available for most subjects. Therefore, HIV was detected by thin-section PCR using DNA extracted from paraffin-embedded cervical cancer specimens as previously described [7]. Briefly, DNA extracted from paraffin blocks was used to amplify a 142 base pair segment of the HIV-1 gag gene (nucleotides 1360-1507) using primers modified from An, SF et al [9]. Primer sequences were SK145-M (5’-GGG ACA TCA AGC CAT GCA AAT-3’) and SK431-M (5’-TGC TAT GTC AGT TCC CCT TGG TTC TCT-3’). A plasmid containing the complete gag and pol genes (pMDLg/pRRE) was used as a positive control (provided by Kenneth Cornetta, Indiana University School of Medicine, Indianapolis, IN). For PCR, 200 ng DNA template was mixed with 25 μL of Master Mix FastStart kit (Roche), 3 μM downstream and upstream primers, and distilled water for a total volume of 50 μL. Reactions were subjected to 1 cycle at 94 °C for 9 min, then 40 cycles at 94 °C for 30 s (denaturation), 55 °C for 30 s (annealing), 72 °C for 30 s (extension), followed by 1 cycle at 72 °C for 7 min (final extension) in a Mastercycler ProS (Eppendorf, Hamburg, Germany).

HIV PCR products were analyzed on 1.8 % agarose gels. To confirm the amplification of desired products, two reactions from cancers with an amplicon of expected size were extracted from gels using the QIAquick Gel Extraction Kit (Qiagen) and sequenced bi-directionally. Homology of the sequenced samples with known HIV gag gene sequences was verified using the Basic Local Sequencing Alignment Search Tool (BLAST) search (NCBI).

### Statistical analysis

This was a comparative study, not a clinic trial, as specimens were archived. Adequacy of extracted DNA was based on β-globin-positivity among ICC specimens. ICC specimens positive for the β-globin human control gene in the Roche Linear Array Assay were included in the analysis of HPV types. HPV type-specific prevalence (oncogenic, or “high-risk” (HR types) and non-oncogenic, or “low-risk (LR) types) was compared among β-globin-positive ICCs from the three countries, and between specimens that were from HIV-infected or HIV-uninfected women from Kenya or Botswana. In addition, HPV types were divided by their respective classification into Alpha 9 (A9 or HPV 16-related) and Alpha 7 (A7 or HPV 18-related). The A9 types included HPV 16, 31, 33, 35, 52, and 58, and A7 types included HPV 18, 39, 45, 59, and 68. Prevalence of A9 and A7 types between countries were compared using Fisher’s exact tests or Chi-square tests. Analyses were performed using SAS (Version 9.3, SAS Institute Inc., Cary, NC).

## Results

### Study specimens

Paraffin-embedded tissue blocks were previously obtained from 51 women from the U.S. [7] (Table 1). All 51 specimens from the U.S. met criteria for tissue adequacy based on the amount of epithelium present. From Botswana, paraffin-embedded tissue blocks were previously obtained from 214 women; 182 (85.0 %) met adequacy criteria [7]. From Kenya, paraffin-embedded tissue blocks were obtained from 210 women; 187 (89.0 %) met adequacy criteria. Fewer specimens from Botswana or Kenya contained a sufficient amount of epithelium compared to specimens from the U.S.; this difference was significant (*p* = 0.01).

**Table 1:** Characteristics of invasive cervical cancers from the U.S., Botswana, and Kenya

Specimen characteristics	U.S.	Botswana	Kenya	*p*-value
Specimens obtained	51	214	210	
Number of specimens with sufficient epithelium^a^	51 (100 %)	182 (85.0 %)	187 (89.0 %)	0.01
β-globin-positive (of all specimens with sufficient epithelium)	50 (98.0 %)	171 (94.0 %)	178 (95.2 %)	0.491
HPV-positive (of β-globin-positive specimens)	46 (92 %)	136 (79.5 %)	146 (82.0 %)	0.128

^a^See text (Methods) for explanation

### HPV detection and HPV types in ICC specimens

Amplifiable DNA, as determined by amplification of the human ß-globin target, was extracted from 50 of 51 (98.0 %) adequate specimens from the U.S., 171 of 182 (94.0 %) from Botswana, and 178 of 187 (95.2 %) from Kenya (*p* = 0.49) (Table 1). HPV DNA of any type was identified in 46 of 50 (92.0 %) U.S. specimens with amplifiable DNA, 136 of 171 (79.5 %) specimens from Botswana, and 146 of 178 (82.0 %) Kenyan specimens (*p* = 0.128) (Table 1).

HR-HPV types were detected in 46 of 46 (100 %) HPV-positive specimens from the U.S., 130 of 136 (95.6 %) from Botswana, and 145 of 146 (99.3 %) from Kenya (*p* = 0.085) (Table 2). LR-HPV types were detected in 0 of 46 (0.0 %) of HPV-positive specimens from the U.S., 12 of 136 (8.8 %) from Botswana, and 3 of 146 (2.1 %) from Kenya (*p* = 0.007) (Table 2).

**Table 2:** HPV types in HPV-positive invasive cervical cancers from the U.S., Botswana, and Kenya

HPV type	U.S., *N* = 46	Botswana, *N* = 136	Kenya, *N* = 146	*p*-value
		High-risk (HR) type distribution		
HR-HPV	46 (100 %)	130 (95.6 %)	145 (99.3 %)	0.085
HPV 16	40 (87.0 %)	58 (42.7 %)	118 (80.8 %)	<0.001
HPV 18	4 (8.7 %)	32 (23.5 %)	28 (19.2 %)	0.089
HPV 26	0	5 (3.7 %)	0	0.033
HPV 31	1 (2.2 %)	5 (3.7 %)	2 (1.4 %)	0.430
HPV 33	1 (2.2 %)	7 (5.2 %)	2 (1.4 %)	0.143
HPV 35	0	8 (5.9 %)	1 (0.7 %)	0.021
HPV 39	0	6 (4.4 %)	1 (0.7 %)	0.085
HPV 45	3 (6.5 %)	12 (8.8 %)	9 (6.2 %)	0.676
HPV 51	0	3 (2.2 %)	0	0.123
HPV 52	0	4 (2.9 %)	0	0.081
HPV 53	0	1 (0.7 %)	0	0.555
HPV 56	0	0	0	-
HPV 58	0	2 (1.5 %)	2 (1.4 %)	1.000
HPV 59	0	2 (1.5 %)	2 (1.4 %)	1.000
HPV 66	0	4 (2.9 %)	0	0.081
HPV 67	0	1 (0.7 %)	0	0.555
HPV 68	0	2 (1.5 %)	0	0.432
HPV 69	0	1 (0.7 %)	0	0.555
HPV 70	0	0	0	-
HPV 73	0	0	1 (0.7 %)	1.000
HPV 82	0	2 (1.5 %)	2 (1.4 %)	1.000
HPV IS39	0	0	0	-
		Low-risk (LR) type distribution		
LR-HPV	0	12 (8.8 %)	3 (2.1 %)	0.007
HPV 6	0	1 (0.7 %)	0	0.555
HPV 11	0	1 (0.7 %)	3 (2.1 %)	0.795
HPV 40	0	1 (0.7 %)	0	0.555
HPV 42	0	0	0	-
HPV 54	0	0	0	-
HPV 55	0	1 (0.7 %)	0	0.555
HPV 61	0	0	0	-
HPV 62	0	0	0	-
HPV 71	0	0	0	-
HPV 72	0	0	0	-
HPV 81	0	1 (0.7 %)	0	0.555
HPV 83	0	2 (1.5 %)	0	0.432
HPV 84	0	6 (4.4 %)	0	0.017

HPV 16 was the most frequently detected type in ICC specimens from all three countries, detected in 40 of 46 (87.0 %) HPV-positive specimens from the U.S., 58 of 136 (42.7 %) from Botswana, and 118 of 146 (80.8 %) from Kenya (*P* < 0.001) (Table 2). Next in frequency was HPV 18, detected in 4 of 46 (8.7 %) HPV-positive specimens from the U.S., 32 of 136 (23.5 %) from Botswana, and 28 of 146 (19.2 %) from Kenya (*p* = 0.089) (Table 2). Together, HPV types 16 and/or 18 were identified in 43 of 46 (93.5 %) HPV-positive specimens from the U.S., 84 of 136 (61.8 %) from Botswana, and 137 of 146 (93.8 %) from Kenya (*p* < 0.0001) (not shown). Other HPV types detected in ICC specimens are shown in Table 2.

Types other than HPV 16 and 18 (non-HPV 16/18 types) were detected in 5 of 46 (10.9 %) HPV-positive specimens from the U.S., 65 of 136 (47.8 %) from Botswana, and 25 of 146 (17.1 %) from Kenya (*p* < 0.001) (Table 3). Non-HPV 16/18 HR types were detected in 5 of 46 (10.9 %) HPV-positive specimens from the U.S., 56 of 136 (41.2 %) from Botswana, and 22 of 146 (15.1 %) from Kenya (*p* < 0.001) (Table 3).

**Table 3:** Distribution of groups of HPV types, Alpha 9 and Alpha 7 types in HPV-positive ICC specimens

	U.S. (*N* = 46)	Botswana (*N* = 136)	Kenya (*N* = 146)	*p*-value
All non-HPV 16/18 types	5 (10.9 %)	65 (47.8 %)	25 (17.1 %)	<0.001
Non-HPV 16/18 HR types	5 (10.9 %)	56 (41.2 %)	22 (15.1 %)	< 0.001
A9 Types^a^	41 (89.1 %)	79 (58.1 %)	120 (82.2 %)	< 0.001
HPV 16	40 (87.0 %)	58 (42.7 %)	118 (80.8 %)	< 0.001
Non-16 A9 Types	2 (4.4 %)	26 (19.1 %)	7 (4.8 %)	0.002
A7 Types^b^	7 (15.2 %)	49 (36.0 %)	40 (27.4 %)	0.022
HPV 18	4 (8.7 %)	32 (23.5 %)	28 (19.2 %)	0.089
Non-18 A7 Types	3 (6.5 %)	18 (13.2 %)	12 (8.2 %)	0.259

^a^A9 types = HPV types 16, 31, 33, 35, 52, and 58

^b^A7 types = HPV types 18, 39, 45, 59, and 68

A9 HPV types were detected in 41 of 46 (89.1 %) HPV-positive specimens from the U.S., 79 of 136 (58.1 %) from Botswana, and 120 of 146 (82.2 %) from Kenya (*p* < 0.001) (Table 3). Non-HPV 16 A9 types (HPV 31, 33, 35, 52, and 58) were detected in 2 of 46 (4.4 %) HPV-positive specimens from the U.S., 26 of 136 (19.1 %) from Botswana, and 7 of 146 (4.8 %) from Kenya (*p* = 0.002) (Table 3).

A7 HPV types were detected in 7 of 46 (15.2 %) HPV-positive specimens from the U.S., 49 of 136 (36.0 %) from Botswana, and 40 of 146 (27.4 %) from Kenya (*p* = 0.022) (Table 3). Non-HPV 18 A7 types (39, 45, 59, and 68) were detected in 3 of 46 (6.5 %) HPV-positive specimens from the U.S., 18 of 136 (13.2 %) from Botswana, and 12 of 146 (8.2 %) from Kenya (*p* = 0.259) (Table 3).

### HIV detection and HPV type distribution in ICC specimens

By thin-section PCR, HIV sequences were detected in 2 of 50 (4.0 %) β-globin-positive specimens from the U.S., 54 of 171 (31.6 %) from Botswana, and 55 of 178 (30.9 %) from Kenya (*p* = 0.0003). Looking at only those specimens in which an HPV type was detected, HIV sequences were detected by thin-section PCR in 1 of 46 (2.2 %) specimens from the U.S., 44 of 136 (32.4 %) from Botswana, and 46 of 146 (31.5 %) from Kenya (*p* = 0.0002). As we had previously done for ICCs from women in Botswana, an analysis was performed on ICCs from Kenyan women in which an HPV type was detected to determine if there were differences between the HPV types in cases from HIV-infected women and HIV-uninfected women. No differences in oncogenic HPV types or groups of oncogenic HPV types were found between ICC cases from HIV-infected or HIV-uninfected Kenyan women (Additional file 1). This was in contrast to ICCs from women living in Botswana, as we previously reported, in which HPV 31 was detected in 1 of 92 (1.1 %) ICCs from HIV-uninfected women and 4 of 44 (9.1 %) ICCs from HIV-infected women.

### Distribution of single HPV type vs. multiple HPV type infections in ICC specimens

For all three countries combined, a single HPV type was identified in 279 of 328 (85.1 %) ICC specimens that contained any HPV type. There was no significant difference in the percentage of ICC specimens containing more than a single HPV type between the three countries (data not shown). In addition, because HIV infection has been associated with detection of multiple HPV types in clinical specimens in some studies, an analysis was performed to determine if detection of multiple HPV types was more common in cancers from HIV-infected women compared to HIV-uninfected women. In ICC specimens from the U.S., Botswana, and Kenya, or the three countries combined, there was no significant association between HIV status and the detection of multiple HPV types (data not shown).

### Theoretical protection against ICC afforded by HPV vaccines

Three HPV vaccines are currently available in the United States: the bivalent vaccine (Cervarix, GSK), the quadrivalent vaccine (Gardasil, Merck), and a recently approved nine-valent vaccine (Gardasil 9, Merck). All three provide excellent protection against HPV 16 and 18. The bivalent and quadrivalent vaccines may each provide a degree of “cross protection” against non-vaccine HR-HPV types, but this is likely to be weak and short-lived compared to efficacy against the oncogenic types actually represented in the vaccines [10]. In contrast, the new nine-valent vaccine provides a high degree of specific protection against infection and disease caused by HR-HPV types 31, 33, 45, 52, and 58, in addition to protection against HPV 16 and HPV 18 [11].

Theoretical vaccine coverage by the currently available HPV vaccines were calculated based on HPV types identified in the ICCs from the three countries (Table 4). The bivalent and quadrivalent vaccines were assumed to be 100 % protective against HPV types 16 and 18. This analysis showed that for the U.S. (Indiana) and Kenya (western Kenya), the bivalent and quadrivalent vaccines should protect against a high percentage of ICC cases (Table 4). In contrast, due to the number of non-HPV 16/18 types identified in ICCs from Botswana, the bivalent and quadrivalent vaccines may provide less protection in that country (Table 4). The percentages of ICCs theoretically covered by the quadrivalent vaccine were 93.5, 61.8, and 93.9 %. The nine-valent vaccine would theoretically provide a higher degree of protection against ICC among women living in any of the three countries, but especially for women living in Botswana (Table 4). The percentages of ICCs theoretically covered by the nine-valent vaccine were 100, 77.8, and 98.0 % in ICCs from the U.S., Botswana, and Kenya, respectively.

**Table 4:** Theoretical protection of HPV vaccines against invasive cervical cancer in the U.S., Botswana, or Kenya, based on data in the current analysis, assuming 100 % vaccine efficacy against oncogenic HPV types represented in the vaccines. The bivalent vaccine is represented by “2X”, the quadrivalent vaccine by “4X”, and the nine-valent vaccine by “9X”

	U.S.	Botswana	Kenya
2X/4X vaccine	93.5 %	61.8 %	93.9 %
9X vaccine	100 %	77.8 %	98 %

## Discussion

It is important to understand differences in the HPV types causing ICC in different regions of the world, and differences in the HPV types causing ICC between women who are infected or not infected with HIV. This information is paramount in the fight against cervical cancer, especially in the era of safe and effective vaccines against HPV. It is entirely possible that HPV type distributions could differ in ICC cases between different countries in a large continent such as Africa. In addition, because there are several available methods for both collection of cancer cells/tissue and for HPV analysis, comparing studies can be difficult.

More country-specific data is needed before conclusions can be drawn. Two analyses of studies performed in African women suggested that HPV 16 was present in approximately half of ICCs, the lowest prevalence among the major regions of the world [12]. Our own previous study of ICCs obtained from women in Botswana (and included in this study) utilized thin section PCR of actual ICC tissue. DNA was directly extracted from sections of ICC and analyzed using the Roche Linear Array assay. The analysis demonstrated a prevalence of 42.7 % for HPV 16, compared to 87 % of ICCs from the U.S. [7].

We wanted to expand the analysis, using identical methods of tissue collection and HPV typing to a second sub-Saharan country, Kenya. The current study was therefore designed to compare and contrast HPV types causing ICC in the U.S., Botswana, and Kenya. Using a direct approach known as thin-section PCR, we found that the distribution of HPV types in ICC specimens from western Kenyan women was similar to that in cancers from the U.S. No statistically significant differences were found for individual HPV types or for groups of types. HPV 16 was the most frequently detected type in ICCs from women living in the U.S. (87.0 %) and from women living in Kenya (82.0 %). Together, HPV types 16 or 18 were detected in approximately 94 % of ICC specimens from both the U.S. and Kenya.

In contrast, the differences between HPV types in ICCs from women living in Kenya and Botswana are striking and likely not exclusively related to HIV infection prevalence. Fewer ICCs from women living in Botswana contained HPV 16. More non-HPV 16/18 oncogenic types were identified in ICCs from women living in Botswana compared to those from the U.S. or Kenya.

Other groups have also examined ICC cases from sub-Saharan African women, but in each case, different methods were utilized in both sampling and in HPV detection, compared to our study and to each other. Several other studies have included data on HIV status as well, but utilized serological HIV testing, as such information was available. For example, De Vuyst et al., conducted a study of Kenyan women with ICC [13]. Fifty-one HIV-infected women were matched by age to 153 HIV-uninfected women. Exfoliated cervical cells from women with ICC were collected and tested for HPV DNA using the PCR-based SPF10-INNO-LiPA system. The actual tumor was apparently not sampled in this study. Multiple-type infections were more frequent in HIV-infected (37.2 %) than in HIV-uninfected (13.7 %) women, but the overall distribution of HPV types was similar. HPV16 was detected in 41.2 % versus 43.8 % and HPV16 and/or 18 in 64.7 % versus 60.1 % of HIV-infected versus HIV-uninfected women, respectively.

Another study from this group was performed to determine associations of HPV infection and cervical intraepithelial neoplasia (CIN) among 498 HIV-infected Kenyan women who underwent HPV PCR-based testing, cytology, and cervical biopsy [14]. Overall, 68.7 % of women were HPV-positive, 52.6 % with HR-HPV, and 40.2 % had multiple type infections. The most frequent types in 113 CIN2/3 cases were HPV 16 (26.5 %), HPV 35 (19.5 %), and HPV 58 (12.4 %). CD4 count was negatively associated with detection of HR-HPV (*P* < 0.001) and CIN2/3 among women not receiving anti-retroviral treatment (*P* = 0.013). The authors concluded that the burden of HR-HPV and CIN2/3 was high in HIV-infected Kenyan women and was related to immunosuppression level. A third study from that group examined frozen tissue biopsies from women with ICC in Kenya and South Africa [15]. One hundred and six HIV-infected and 129 HIV-uninfected women with ICC were included. A fragment from the biopsy was tested for HPV DNA using GP5+/6 + -PCR assay Multiple HPV types were identified in 21.6 % of ICCs from HIV-infected women compared 3.3 % in HIV-uninfected women. HPV16 and/or 18 prevalence combined was similar in ICCS from HIV-infected (66.7 %) and HIV-uninfected women (69.1 %) No significant difference was found for other HPV types between the two groups. The authors concluded that current prophylactic HPV vaccines against HPV 16 and 18 may prevent similar proportions of cervical cancers in HIV-infected and HIV-uninfected Kenyan women.

A recent study by Maranga et al., examined HPV types in cervical smears and carcinomas from HIV-infected and HIV-uninfected Kenyan women [16]. Smear samples from women were analyzed, and those from HIV-infected women had a higher rate of detection of HR-HPV types 52, 58, 68, potential high risk 53/70, and low-risk 44/55 compared to HIV-uninfected women. HPV types 16/18 were detected in cells collected from ~46 % of HIV-infected women and ~46 % of HIV-positive women. HPV 45 was more common in cells collected from HIV-infected women. The authors concluded that HIV infection may alter the spectrum of HPV types found in cervical smears and in ICC.

HIV co-infection accelerates the natural history of cervical cancer. For Kenya and Botswana, we did not know the HIV status for the women. Using a thin-section PCR devised by our group, HIV was identified in ~32 to 34 % of ICC specimens from women living in Botswana or Kenyan, compared to ~4 % of ICCs from women living in Indiana. These numbers do not reflect the HIV prevalence of all women in these countries but illustrate the higher incidence of ICC in HIV-infected women. More studies of the thin-section PCR for HIV are needed to validate this potentially useful assay. Based on studies by other groups, we expected that ICCs from women who were HIV-infected may have a different HPV type distribution that from ICCs from HIV-uninfected women in these countries [17]. However, when ICC cases were stratified by HIV status, no significant differences were found for any HPV type or groups of types. The reason for this observation is not clear, and further study is needed.

The current study has implications for health policies related to prevention of cervical cancer. This study indicates that HPV types 16 and 18 are the primary types causing ICC in Indiana, U.S., and in western Kenya. However, our recent study in Botswana (and shown again in this manuscript) showed a different HPV type distribution. As indicated above, three HPV vaccines are currently available: the bivalent vaccine, the quadrivalent vaccine, and the nine-valent vaccine. All are expensive, costing approximately $360 in the U.S. for the series of three required doses. All three provide excellent protection against HPV 16 and 18. The nine-valent HPV vaccine includes VLPs present in the quadrivalent vaccine (HPV types 6, 11, 16, and 18) plus VLPs of five additional HR-HPV types (31, 33, 45, 52, and 58) [11].

When global data is examined, the five additional HR-HPV types in the nine-valent HPV vaccine account for approximately 12 % of all cervical cancers [11]. Combined with HPV 16 and HPV 18 estimates, the nine-valent HPV vaccine has the potential to protect against nine types responsible for greater than 90 % of cervical cancers. The U.S. Food and Drug Administration (FDA) recently approved the nine-valent vaccine for use. A cost-benefit analysis would be required to determine if the newer vaccine would be worth any additional cost, in terms of life-years gained compared to the other available HPV vaccines.

How important would the nine-valent HPV vaccine be in Botswana? Due to the large percentage of non-HPV 16/18 types identified in the ICC cases in Botswana, the bivalent and quadrivalent vaccines would theoretically provide less protection against ICC than it would for women in Kenya or the U.S. The nine-valent vaccine would theoretically provide a higher degree of protection against ICC among women living in Botswana.

There are several limitations to this study. First, only ICC cases from western Kenya were included and results cannot be generalized to the entire country of Kenya. Second, this study used paraffin-embedded cervical cancers, and as a result, the sensitivity for HPV detection could be less than if fresh cancer tissues had been available. Because the current study used archived specimens, fixation conditions could not be controlled and may have contributed to differences between countries in the frequencies of HPV detection [18]. Third, it is possible that the techniques used to recover DNA and detect/type HPV have differing capabilities for certain HPV types, thus potentially underrepresenting non-HPV 16/18 HPV types. However, the Roche Linear Array is the standard detection and typing method in epidemiologic studies of HPV, and in other studies has demonstrated the capacity to detect numerous HPV types in cervical cancers [19]. Fourth, only 85 to 89 % of the samples from African women were analyzed due to inadequate epithelium histologically, and the inability to identify HPV in roughly 20 % of the African specimens could shift the results and conclusions, given the small size of the study. Fifth, the proportion of HPV-negative cancers in Botswana (among those with adequate beta-globin DNA) is curious and could be related to differences in fixation and processing techniques as mentioned above. It is possible that HPV types in some of these ICCs are not detected in the Linear Array Assay. Sixth, previous studies detected HPV 16 in ~70 to 80 % of ICC cases, compared to >90 % in our study for the ICCs from the U.S. and Kenya. While it is possible that our methods overestimate HPV 16/18 prevalence in ICCs, there is no evidence to support this. Most previous studies used cervical swabs rather than thin-section PCR to capture tumor cells and to determine HPV types in the cancers but this could lead to an overestimation of the role of certain HPV types when compared to thin-section PCR performed directly on ICCs. Lastly, the HIV status of women in Kenya and Botswana was not known. Therefore, an indirect method a thin-section PCR was developed to determine the HIV status of these women. The sensitivity and specificity of this novel method has not yet been determined, but we verified the authenticity of HIV sequences in two ICC specimens.

## Conclusions

Continued efforts must be made to improve utilization of the HPV vaccines, and with the help of the World Health Organization and other agencies, and hopefully a reduced price of the nine-valent HPV vaccine can be negotiated for countries in which non-HPV 16/18 types are frequently identified in careful studies of ICC. For now, every effort should be made to vaccinate young girls and adolescent women with any of the currently available HPV vaccines. This will help assure a cervical cancer-free future for Kenya, Botswana, and other sub-Saharan countries where cervical cancer screening is not widely available.

## Additional file

Additional file 1: Comparison HPV between HIV positive and negative sample from Kenya. (DOCX 50 kb)

## Abbreviations

ASR: Age standardized rate
BLAST: Basic local sequencing alignment search tool
CIN: Cervical intraepithelial neoplasia
FDA: Food and Drug Administration
HIV: Human immunodeficiency virus
HPV: Human papillomavirus
HR: High risk
ICC: Invasive cervical cancer
LR: Low-risk
PCR: Polymerase chain reaction
