# Supplementary material for: Invasive cervical cancers in the United States, Botswana and Kenya: HPV type distribution and health policy implications
Source: Infect Agent Cancer. 2016 Nov 11;11:56. doi: 10.1186/s13027-016-0102-9 (PMC5105291; doi:10.1186/s13027-016-0102-9)
Supplement: Additional file 1: — Comparison HPV between HIV positive and negative sample from Kenya. (DOCX 50 kb) [file 13027_2016_102_MOESM1_ESM.docx]

**Comparison HPV between HIV positive and negative sample from Kenya**

| Table of P16 by HIV | | | |
| --- | --- | --- | --- |
| P16(P16) | HIV(HIV) | | |
| Frequency Col Pct | 0 | 1 | Total |
| 0 | 18 18.00 | 10 21.74 | 28 |
| 1 | 82 82.00 | 36 78.26 | 118 |
| Total | 100 | 46 | 146 |

| Statistic | DF | Value | Prob |
| --- | --- | --- | --- |
| Chi-Square | 1 | 0.2842 | 0.5940 |

| Sample Size = 146 |
| --- |

| Table of P18 by HIV | | | |
| --- | --- | --- | --- |
| P18(P18) | HIV(HIV) | | |
| Frequency Col Pct | 0 | 1 | Total |
| 0 | 80 80.00 | 38 82.61 | 118 |
| 1 | 20 20.00 | 8 17.39 | 28 |
| Total | 100 | 46 | 146 |

| Statistic | DF | Value | Prob |
| --- | --- | --- | --- |
| Chi-Square | 1 | 0.1383 | 0.7099 |

| Sample Size = 146 |
| --- |

| Table of P26 by HIV | | | |
| --- | --- | --- | --- |
| P26(P26) | HIV(HIV) | | |
| Frequency Col Pct | 0 | 1 | Total |
| 0 | 100 100.00 | 46 100.00 | 146 |
| Total | 100 | 46 | 146 |

| Table of P31 by HIV | | | |
| --- | --- | --- | --- |
| P31(P31) | HIV(HIV) | | |
| Frequency Col Pct | 0 | 1 | Total |
| 0 | 99 99.00 | 45 97.83 | 144 |
| 1 | 1 1.00 | 1 2.17 | 2 |
| Total | 100 | 46 | 146 |

| Fisher's Exact Test | |
| --- | --- |
| Two-sided Pr <= P | 0.5324 |

| Sample Size = 146 |
| --- |

| Table of P33 by HIV | | | |
| --- | --- | --- | --- |
| P33(P33) | HIV(HIV) | | |
| Frequency Col Pct | 0 | 1 | Total |
| 0 | 99 99.00 | 45 97.83 | 144 |
| 1 | 1 1.00 | 1 2.17 | 2 |
| Total | 100 | 46 | 146 |

| Fisher's Exact Test | |
| --- | --- |
| Two-sided Pr <= P | 0.5324 |

| Sample Size = 146 |
| --- |

| Table of P35 by HIV | | | |
| --- | --- | --- | --- |
| P35(P35) | HIV(HIV) | | |
| Frequency Col Pct | 0 | 1 | Total |
| 0 | 99 99.00 | 46 100.00 | 145 |
| 1 | 1 1.00 | 0 0.00 | 1 |
| Total | 100 | 46 | 146 |

| Fisher's Exact Test | |
| --- | --- |
| Two-sided Pr <= P | 1.0000 |

| Sample Size = 146 |
| --- |

| Table of P39 by HIV | | | |
| --- | --- | --- | --- |
| P39(P39) | HIV(HIV) | | |
| Frequency Col Pct | 0 | 1 | Total |
| 0 | 100 100.00 | 45 97.83 | 145 |
| 1 | 0 0.00 | 1 2.17 | 1 |
| Total | 100 | 46 | 146 |

| Fisher's Exact Test | |
| --- | --- |
| Two-sided Pr <= P | 0.3151 |

| Sample Size = 146 |
| --- |

| Table of P45 by HIV | | | |
| --- | --- | --- | --- |
| P45(P45) | HIV(HIV) | | |
| Frequency Col Pct | 0 | 1 | Total |
| 0 | 93 93.00 | 44 95.65 | 137 |
| 1 | 7 7.00 | 2 4.35 | 9 |
| Total | 100 | 46 | 146 |

| Fisher's Exact Test | |
| --- | --- |
| Two-sided Pr <= P | 0.7199 |

| Sample Size = 146 |
| --- |

| Table of P51 by HIV | | | |
| --- | --- | --- | --- |
| P51(P51) | HIV(HIV) | | |
| Frequency Col Pct | 0 | 1 | Total |
| 0 | 100 100.00 | 46 100.00 | 146 |
| Total | 100 | 46 | 146 |

| Table of P52 by HIV | | | |
| --- | --- | --- | --- |
| P52(P52) | HIV(HIV) | | |
| Frequency Col Pct | 0 | 1 | Total |
| 0 | 100 100.00 | 46 100.00 | 146 |
| Total | 100 | 46 | 146 |

| Table of P53 by HIV | | | |
| --- | --- | --- | --- |
| P53(P53) | HIV(HIV) | | |
| Frequency Col Pct | 0 | 1 | Total |
| 0 | 100 100.00 | 46 100.00 | 146 |
| Total | 100 | 46 | 146 |

| Table of P56 by HIV | | | |
| --- | --- | --- | --- |
| P56(P56) | HIV(HIV) | | |
| Frequency Col Pct | 0 | 1 | Total |
| 0 | 100 100.00 | 46 100.00 | 146 |
| Total | 100 | 46 | 146 |

| Table of P58 by HIV | | | |
| --- | --- | --- | --- |
| P58(P58) | HIV(HIV) | | |
| Frequency Col Pct | 0 | 1 | Total |
| 0 | 99 99.00 | 45 97.83 | 144 |
| 1 | 1 1.00 | 1 2.17 | 2 |
| Total | 100 | 46 | 146 |

| Fisher's Exact Test | |
| --- | --- |
| Two-sided Pr <= P | 0.5324 |

| Sample Size = 146 |
| --- |

| Table of P59 by HIV | | | |
| --- | --- | --- | --- |
| P59(P59) | HIV(HIV) | | |
| Frequency Col Pct | 0 | 1 | Total |
| 0 | 98 98.00 | 46 100.00 | 144 |
| 1 | 2 2.00 | 0 0.00 | 2 |
| Total | 100 | 46 | 146 |

| Fisher's Exact Test | |
| --- | --- |
| Two-sided Pr <= P | 1.0000 |

| Sample Size = 146 |
| --- |

| Table of P66 by HIV | | | |
| --- | --- | --- | --- |
| P66(P66) | HIV(HIV) | | |
| Frequency Col Pct | 0 | 1 | Total |
| 0 | 100 100.00 | 46 100.00 | 146 |
| Total | 100 | 46 | 146 |

| Table of P67 by HIV | | | |
| --- | --- | --- | --- |
| P67(P67) | HIV(HIV) | | |
| Frequency Col Pct | 0 | 1 | Total |
| 0 | 100 100.00 | 46 100.00 | 146 |
| Total | 100 | 46 | 146 |

| Table of P68 by HIV | | | |
| --- | --- | --- | --- |
| P68(P68) | HIV(HIV) | | |
| Frequency Col Pct | 0 | 1 | Total |
| 0 | 100 100.00 | 46 100.00 | 146 |
| Total | 100 | 46 | 146 |

| Table of P69 by HIV | | | |
| --- | --- | --- | --- |
| P69(P69) | HIV(HIV) | | |
| Frequency Col Pct | 0 | 1 | Total |
| 0 | 100 100.00 | 46 100.00 | 146 |
| Total | 100 | 46 | 146 |

| Table of P70 by HIV | | | |
| --- | --- | --- | --- |
| P70(P70) | HIV(HIV) | | |
| Frequency Col Pct | 0 | 1 | Total |
| 0 | 100 100.00 | 46 100.00 | 146 |
| Total | 100 | 46 | 146 |

| Table of P73 by HIV | | | |
| --- | --- | --- | --- |
| P73(P73) | HIV(HIV) | | |
| Frequency Col Pct | 0 | 1 | Total |
| 0 | 100 100.00 | 45 97.83 | 145 |
| 1 | 0 0.00 | 1 2.17 | 1 |
| Total | 100 | 46 | 146 |

| Fisher's Exact Test | |
| --- | --- |
| Two-sided Pr <= P | 0.3151 |

| Sample Size = 146 |
| --- |

| Table of P82 by HIV | | | |
| --- | --- | --- | --- |
| P82(P82) | HIV(HIV) | | |
| Frequency Col Pct | 0 | 1 | Total |
| 0 | 99 99.00 | 45 97.83 | 144 |
| 1 | 1 1.00 | 1 2.17 | 2 |
| Total | 100 | 46 | 146 |

| Fisher's Exact Test | |
| --- | --- |
| Two-sided Pr <= P | 0.5324 |

| Sample Size = 146 |
| --- |

| Table of IS39 by HIV | | | |
| --- | --- | --- | --- |
| IS39(IS39) | HIV(HIV) | | |
| Frequency Col Pct | 0 | 1 | Total |
| 0 | 100 100.00 | 46 100.00 | 146 |
| Total | 100 | 46 | 146 |

| Table of P6 by HIV | | | |
| --- | --- | --- | --- |
| P6(P6) | HIV(HIV) | | |
| Frequency Col Pct | 0 | 1 | Total |
| 0 | 100 100.00 | 46 100.00 | 146 |
| Total | 100 | 46 | 146 |

| Table of P11 by HIV | | | |
| --- | --- | --- | --- |
| P11(P11) | HIV(HIV) | | |
| Frequency Col Pct | 0 | 1 | Total |
| 0 | 100 100.00 | 43 93.48 | 143 |
| 1 | 0 0.00 | 3 6.52 | 3 |
| Total | 100 | 46 | 146 |

| Fisher's Exact Test | |
| --- | --- |
| Two-sided Pr <= P | 0.0299 |

| Sample Size = 146 |
| --- |

| Table of P40 by HIV | | | |
| --- | --- | --- | --- |
| P40(P40) | HIV(HIV) | | |
| Frequency Col Pct | 0 | 1 | Total |
| 0 | 100 100.00 | 46 100.00 | 146 |
| Total | 100 | 46 | 146 |

| Table of P42 by HIV | | | |
| --- | --- | --- | --- |
| P42(P42) | HIV(HIV) | | |
| Frequency Col Pct | 0 | 1 | Total |
| 0 | 100 100.00 | 46 100.00 | 146 |
| Total | 100 | 46 | 146 |

| Table of P54 by HIV | | | |
| --- | --- | --- | --- |
| P54(P54) | HIV(HIV) | | |
| Frequency Col Pct | 0 | 1 | Total |
| 0 | 100 100.00 | 46 100.00 | 146 |
| Total | 100 | 46 | 146 |

| Table of P55 by HIV | | | |
| --- | --- | --- | --- |
| P55(P55) | HIV(HIV) | | |
| Frequency Col Pct | 0 | 1 | Total |
| 0 | 100 100.00 | 46 100.00 | 146 |
| Total | 100 | 46 | 146 |

| Table of P61 by HIV | | | |
| --- | --- | --- | --- |
| P61(P61) | HIV(HIV) | | |
| Frequency Col Pct | 0 | 1 | Total |
| 0 | 100 100.00 | 46 100.00 | 146 |
| Total | 100 | 46 | 146 |

| Table of P62 by HIV | | | |
| --- | --- | --- | --- |
| P62(P62) | HIV(HIV) | | |
| Frequency Col Pct | 0 | 1 | Total |
| 0 | 100 100.00 | 46 100.00 | 146 |
| Total | 100 | 46 | 146 |

| Table of P64 by HIV | | | |
| --- | --- | --- | --- |
| P64(P64) | HIV(HIV) | | |
| Frequency Col Pct | 0 | 1 | Total |
| 0 | 100 100.00 | 46 100.00 | 146 |
| Total | 100 | 46 | 146 |

| Table of P71 by HIV | | | |
| --- | --- | --- | --- |
| P71(P71) | HIV(HIV) | | |
| Frequency Col Pct | 0 | 1 | Total |
| 0 | 100 100.00 | 46 100.00 | 146 |
| Total | 100 | 46 | 146 |

| Table of P72 by HIV | | | |
| --- | --- | --- | --- |
| P72(P72) | HIV(HIV) | | |
| Frequency Col Pct | 0 | 1 | Total |
| 0 | 100 100.00 | 46 100.00 | 146 |
| Total | 100 | 46 | 146 |

| Table of P81 by HIV | | | |
| --- | --- | --- | --- |
| P81(P81) | HIV(HIV) | | |
| Frequency Col Pct | 0 | 1 | Total |
| 0 | 100 100.00 | 46 100.00 | 146 |
| Total | 100 | 46 | 146 |

| Table of P83 by HIV | | | |
| --- | --- | --- | --- |
| P83(P83) | HIV(HIV) | | |
| Frequency Col Pct | 0 | 1 | Total |
| 0 | 100 100.00 | 46 100.00 | 146 |
| Total | 100 | 46 | 146 |

| Table of P84 by HIV | | | |
| --- | --- | --- | --- |
| P84(P84) | HIV(HIV) | | |
| Frequency Col Pct | 0 | 1 | Total |
| 0 | 100 100.00 | 46 100.00 | 146 |
| Total | 100 | 46 | 146 |

| Table of AnyHPV by HIV | | | |
| --- | --- | --- | --- |
| AnyHPV | HIV(HIV) | | |
| Frequency Col Pct | 0 | 1 | Total |
| 1 | 100 100.00 | 46 100.00 | 146 |
| Total | 100 | 46 | 146 |

| Table of HRHPV by HIV | | | |
| --- | --- | --- | --- |
| HRHPV | HIV(HIV) | | |
| Frequency Col Pct | 0 | 1 | Total |
| 0 | 0 0.00 | 1 2.17 | 1 |
| 1 | 100 100.00 | 45 97.83 | 145 |
| Total | 100 | 46 | 146 |

| Fisher's Exact Test | |
| --- | --- |
| Two-sided Pr <= P | 0.3151 |

| Sample Size = 146 |
| --- |

| Table of LRHPV by HIV | | | |
| --- | --- | --- | --- |
| LRHPV | HIV(HIV) | | |
| Frequency Col Pct | 0 | 1 | Total |
| 0 | 100 100.00 | 43 93.48 | 143 |
| 1 | 0 0.00 | 3 6.52 | 3 |
| Total | 100 | 46 | 146 |

| Fisher's Exact Test | |
| --- | --- |
| Two-sided Pr <= P | 0.0299 |

| Sample Size = 146 |
| --- |

| Table of HPV1618 by HIV | | | |
| --- | --- | --- | --- |
| HPV1618 | HIV(HIV) | | |
| Frequency Col Pct | 0 | 1 | Total |
| 0 | 4 4.00 | 5 10.87 | 9 |
| 1 | 96 96.00 | 41 89.13 | 137 |
| Total | 100 | 46 | 146 |

| Fisher's Exact Test | |
| --- | --- |
| Two-sided Pr <= P | 0.1406 |

| Sample Size = 146 |
| --- |

| Table of Non1618HPV by HIV | | | |
| --- | --- | --- | --- |
| Non1618HPV | HIV(HIV) | | |
| Frequency Col Pct | 0 | 1 | Total |
| 0 | 86 86.00 | 35 76.09 | 121 |
| 1 | 14 14.00 | 11 23.91 | 25 |
| Total | 100 | 46 | 146 |

| Statistic | DF | Value | Prob |
| --- | --- | --- | --- |
| Chi-Square | 1 | 2.1817 | 0.1397 |

| Sample Size = 146 |
| --- |

| Table of Non1618HRHPV by HIV | | | |
| --- | --- | --- | --- |
| Non1618HRHPV | HIV(HIV) | | |
| Frequency Col Pct | 0 | 1 | Total |
| 0 | 86 86.00 | 38 82.61 | 124 |
| 1 | 14 14.00 | 8 17.39 | 22 |
| Total | 100 | 46 | 146 |

| Statistic | DF | Value | Prob |
| --- | --- | --- | --- |
| Chi-Square | 1 | 0.2831 | 0.5947 |

| Sample Size = 146 |
| --- |

| Table of A9HPV by HIV | | | |
| --- | --- | --- | --- |
| A9HPV | HIV(HIV) | | |
| Frequency Col Pct | 0 | 1 | Total |
| 0 | 17 17.00 | 9 19.57 | 26 |
| 1 | 83 83.00 | 37 80.43 | 120 |
| Total | 100 | 46 | 146 |

| Statistic | DF | Value | Prob |
| --- | --- | --- | --- |
| Chi-Square | 1 | 0.1416 | 0.7067 |

| Sample Size = 146 |
| --- |

| Table of Non16A9HPV by HIV | | | |
| --- | --- | --- | --- |
| Non16A9HPV | HIV(HIV) | | |
| Frequency Col Pct | 0 | 1 | Total |
| 0 | 96 96.00 | 43 93.48 | 139 |
| 1 | 4 4.00 | 3 6.52 | 7 |
| Total | 100 | 46 | 146 |

| Fisher's Exact Test | |
| --- | --- |
| Two-sided Pr <= P | 0.6786 |

| Sample Size = 146 |
| --- |

| Table of A7HPV by HIV | | | |
| --- | --- | --- | --- |
| A7HPV | HIV(HIV) | | |
| Frequency Col Pct | 0 | 1 | Total |
| 0 | 71 71.00 | 35 76.09 | 106 |
| 1 | 29 29.00 | 11 23.91 | 40 |
| Total | 100 | 46 | 146 |

| Statistic | DF | Value | Prob |
| --- | --- | --- | --- |
| Chi-Square | 1 | 0.4099 | 0.5220 |

| Sample Size = 146 |
| --- |

| Table of Non18A7HPV by HIV | | | |
| --- | --- | --- | --- |
| Non18A7HPV | HIV(HIV) | | |
| Frequency Col Pct | 0 | 1 | Total |
| 0 | 91 91.00 | 43 93.48 | 134 |
| 1 | 9 9.00 | 3 6.52 | 12 |
| Total | 100 | 46 | 146 |

| Fisher's Exact Test | |
| --- | --- |
| Two-sided Pr <= P | 0.7533 |

| Sample Size = 146 |
| --- |

| Table of AnyHPVOrg by HIV | | | |
| --- | --- | --- | --- |
| AnyHPVOrg | HIV(HIV) | | |
| Frequency Col Pct | 0 | 1 | Total |
| 1 | 84 84.00 | 39 84.78 | 123 |
| 2 | 16 16.00 | 5 10.87 | 21 |
| 3 | 0 0.00 | 2 4.35 | 2 |
| Total | 100 | 46 | 146 |

| Fisher's Exact Test | |
| --- | --- |
| Pr <= P | 0.0963 |

| Sample Size = 146 |
| --- |
